# P-572. Retrospective Analysis of Vibriosis presenting to a Tertiary University-Based Hospital in Florida and Correlation with Sea Surface Temperatures from 2011 to 2023

**DOI:** 10.1093/ofid/ofaf695.787

**Published:** 2026-01-11

**Authors:** Joshua B French, Sunil Kamar, Antarpreet S Jutla, Norman Beatty

**Affiliations:** University of Florida College of Medicine, Gainesville, FL; University of Florida Department of Environmental Engineering Sciences, Gainesville, Florida; University of Florida Department of Environmental Engineering Sciences, Gainesville, Florida; University of Florida, Gainesville, Florida

## Abstract

**Background:**

Vibrio species are free-living Gram-negative bacteria which are ubiquitous to certain marine and estuarine environments along the coasts of Florida. Vibriosis can take on several clinical forms, including gastrointestinal illnesses (GI) leading to diarrheal disease, skin and soft tissue infections (SSTI) which are known to be necrotizing, and bacteremia with sepsis. The effect of climate change on Vibrio species distribution and human infection is not well studied. We aim to assess whether warmer sea surface temperatures (SSTs) correlate with higher prevalence of Vibriosis.

**Figure 1:**
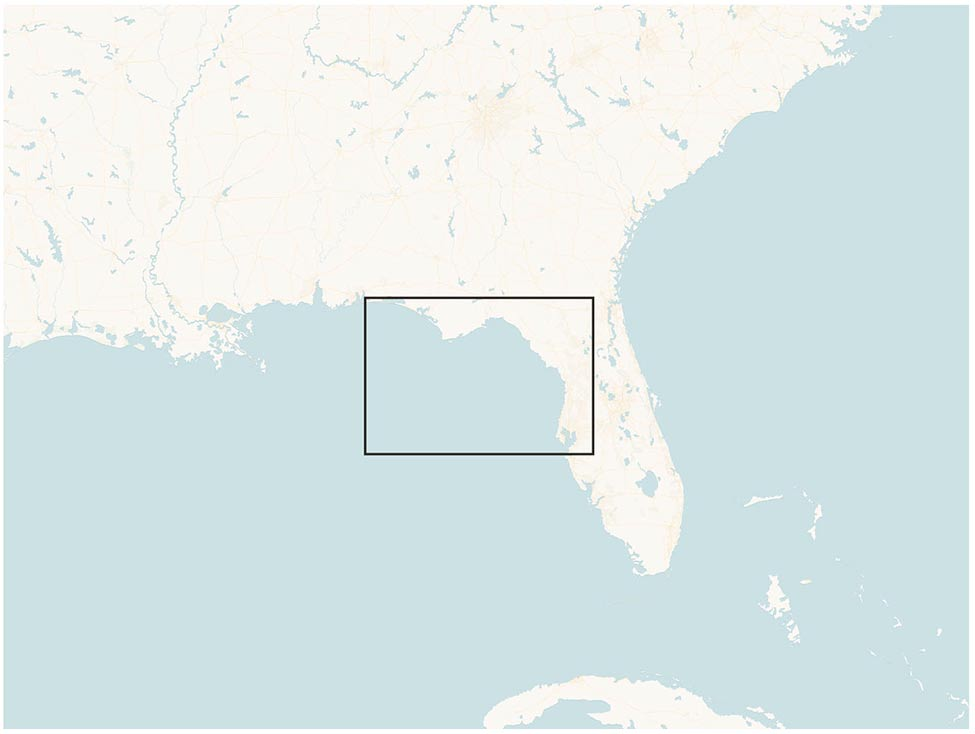
Map of the Gulf of Mexico showing the region used for SST analysis (2011 – 2023). SST data was obtained from the UF Department of Environmental Engineering Sciences, via the NASA Moderate Resolution Imaging Spectroradiometer (MODIS), which provides data at a spatial resolution of 4 km x 4km. The study area reflects coastal waters associated with exposure among the UF Health patient population.

**Methods:**

A retrospective analysis of patients presenting to University of Florida Health Shands Hospital (Gainesville, Florida) from January 1st 2011 until December 31st 2023 was conducted to assess frequency and clinical outcomes of vibriosis. We also compared data from NASA's Aqua-MODIS satellite (Figure 1) with frequency of infection. Inclusion criteria for vibriosis consisted of detection of Vibrio species DNA via PCR technology or conventional microbiological growth from a clinical specimen.

**Figure 2:**
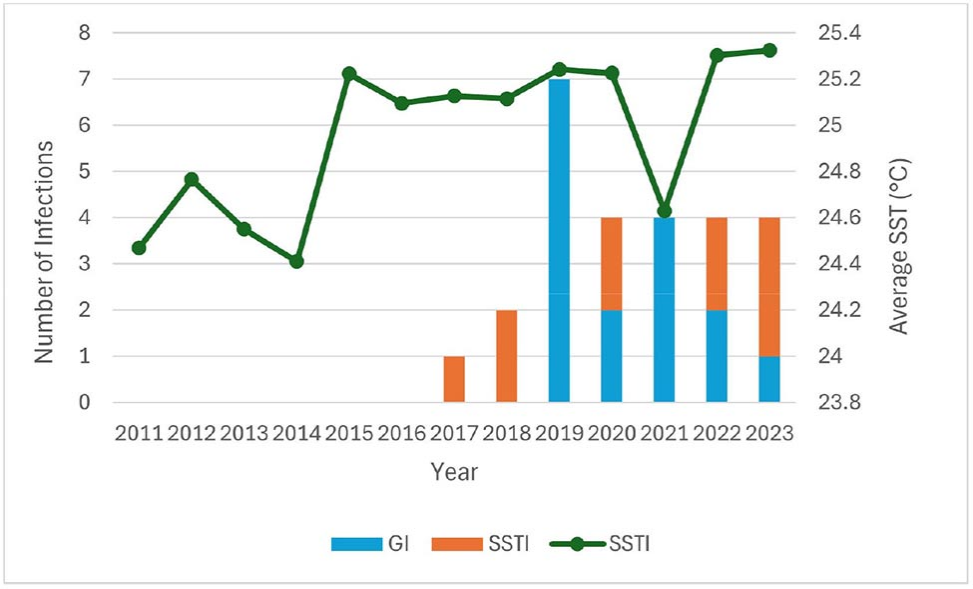
Year of infection by number and type of infection as well as average SST. Infections have increased in recent years, with half of SSTI cases occurring in 2022-2023.

**Results:**

Twenty-six total patients were diagnosed with vibriosis during the study period. From 2011 until 2016 no Vibriosis cases were reported at our hospital. From 2017 until 2023 we averaged 3.7 cases per year, with an average length of stay (LOS) of 10.3 days. Ten (38%) SSTI and sixteen (61%) GI were documented. The majority (n= 9/10) of SSTIs occurred between the months of June to November. No patient deaths were documented because of vibriosis.

The average sea surface temperature (SST) in 2011 was 24.47 °C and has generally risen by about 0.07 °C per year, with the average SST in 2023 being 25.32 °C.

**Conclusion:**

Vibriosis, especially necrotizing SSTIs, can lead to significant morbidity and death. Our hospital located in north central Florida is situated approximately 50-100 miles inland from the Gulf coast, yet we have now started to see consistent Vibriosis cases. Our results suggest there is a correlation between the rise in SST of the Gulf waters and vibriosis cases presenting to our hospital system. Ongoing research is needed to better understand the relationship of rising SST and clinical vibriosis in our state and improve education on this potentially life-threatening infectious process.

**Disclosures:**

All Authors: No reported disclosures

